# Vitamin D and Pancreatitis: A Narrative Review of Current Evidence

**DOI:** 10.3390/nu14102113

**Published:** 2022-05-18

**Authors:** Fei Cai, Cheng Hu, Chan-Juan Chen, Yuan-Ping Han, Zi-Qi Lin, Li-Hui Deng, Qing Xia

**Affiliations:** 1 Department and Laboratory of Integrated Traditional Chinese and Western Medicine, Sichuan Provincial Pancreatitis Centre and West China-Liverpool Biomedical Research Centre, West China Hospital, Sichuan University, Chengdu 610041, China; caifeiscu@163.com (F.C.); hc715@163.com (C.H.); cchanjuan@163.com (C.-J.C.); linziqi_85@163.com (Z.-Q.L.); xiaqing@medmail.com.cn (Q.X.); 2 The Center for Growth, Metabolism and Aging, College of Life Sciences, Sichuan University, Chengdu 610017, China; hanyp@scu.edu.cn

**Keywords:** acute pancreatitis, chronic pancreatitis, vitamin D, vitamin D receptor, vitamin D analog

## Abstract

Emerging research indicates that vitamin D metabolic disorder plays a major role in both acute pancreatitis (AP) and chronic pancreatitis (CP). This has been demonstrated by studies showing that vitamin D deficiency is associated with pancreatitis and its anti-inflammatory and anti-fibrotic effects by binding with the vitamin D receptor (VDR). However, the role of vitamin D assessment and its management in pancreatitis remains poorly understood. In this narrative review, we discuss the recent advances in our understanding of the molecular mechanisms involved in vitamin D/VDR signaling in pancreatic cells; the evidence from observational studies and clinical trials that demonstrate the connection among vitamin D, pancreatitis and pancreatitis-related complications; and the route of administration of vitamin D supplementation in clinical practice. Although further research is still required to establish the protective role of vitamin D and its application in disease, evaluation of vitamin D levels and its supplementation should be important strategies for pancreatitis management according to currently available evidence.

## 1. Introduction

Both acute and chronic pancreatitis are common digestive diseases for which specific treatment is not yet available. Acute pancreatitis (AP) is characterized by sudden onset triggered by various factors such as gallstones, alcoholism, hypertriglyceridaemia, and endoscopic retrograde cholangiopancreatography (ERCP), leading to self-digestion of acinar cells that induce local and systemic inflammation [1]. The prevalence of AP has been increasing, with an overall incidence of >34 affected cases per 100,000 person-years [2]. Most cases resolve within two weeks. However, approximately 10–20% of AP patients have an eventful clinical course, from local pancreatic fluid collection and/or necrosis to critical illness of persistent organ failure with a substantial mortality rate of 20–50% [3]. Emerging evidence indicates that patients with an episode of AP have a 20–30% likelihood of one or more recurrent attacks, with progression to chronic pancreatitis (CP) in an estimated 10% of the recurrent cases [1,4]. Once CP is established, the risk for pancreatic cancer rises by 13.3-fold [5].

Vitamin D refers to a group of steroid hormones that are naturally present in small amounts in food, but it is mostly synthesized endogenously by ultraviolet (UV) rays from sunlight acting on the skin. The biologically inactive precursors, namely vitamin D_2_ (ergocalciferol) and vitamin D_3_ (cholecalciferol), convert into the active compound 1,25(OH)_2_D by two enzymatic hydroxylation reactions after they enter the circulation. Biologically active 1,25(OH)_2_D binds to and stimulates the transcriptional activity of the nuclear vitamin D receptor (VDR) in target cells to regulate the expression of genes, thus altering cellular activities. The main function of vitamin D is to regulate calcium homeostasis and maintain a healthy mineralized skeleton. Vitamin D is also critical for pleiotropic functions such as anti-inflammation, immune regulation, tumor suppression, and metabolic homeostasis [6]. Lack of sunshine exposure; seasonal variation; pregnancy; older age; obesity; and ethnicity (Black, Hispanic, and subjects with increased skin melanin deposition), are particularly high-risk factors for vitamin D deficiency [7,8,9,10].

Emerging studies indicate the association of vitamin D with pancreatitis [11,12]. Most patients who develop pancreatitis have changes in their dietary habits prior to the onset of the disease, either due to heavy alcohol consumption or fat intolerance. Because of maldigestion/malabsorption alone, and complicated with other factors such as a low dietary vitamin uptake, low exposure to sunshine, and exocrine dysfunction, nutritional deficiencies, especially in fat-soluble vitamins (vitamins A, D, E, and K), have been demonstrated in patients with AP and CP. Although nutritional management has been suggested in several guidelines [13,14,15,16,17], the role of vitamin D assessment and its management in pancreatitis remains underestimated by physicians.

## 2. Search Strategy

Two investigators independently performed a systematic computerized search for related articles through MEDLINE (PubMed) and Web of Science from their inception to 1 April 2022. The search strategy used a combination of the following keywords: “vitamin D”, “vitamin D deficiency”, “cholecalciferol”, “vitamin D receptor”, “VDR”, “ergocalciferol”, “pancreatitis”, “chronic pancreatitis”, and “acute pancreatitis”. All studies investigating vitamin D in experimental and clinical exocrine pancreatic diseases were initially included. Possible additional articles were identified by manually searching the reference lists of all the retrieved articles to identify potentially relevant studies. Only studies in English were analyzed. In all, 111 relevant articles were selected and included in the present narrative review.

## 3. Vitamin D Metabolism and Its Biological Actions in Basic Studies

### 3.1. Vitamin D Metabolism

Biologically active vitamin D, 1,25(OH)_2_D (also known as calcitriol), is converted by its precursors vitamin D_2_ (ergocalciferol) from plant-based foods and vitamin D_3_ (cholecalciferol) mostly from animal-based sources or ultraviolet rays (UV, spectrum 290–315 nm) from sunlight [18,19,20]. The synthesis of 1,25(OH)_2_D is mainly regulated by two enzymatic hydroxylation reactions in the liver and kidney. The extrarenal synthesis of 1,25(OH)_2_D is regulated by CYP27B1, which is expressed locally in tissues including the colon, parathyroid, prostate, breast, brain, placenta, and pancreas [21].

**Figure 1:**
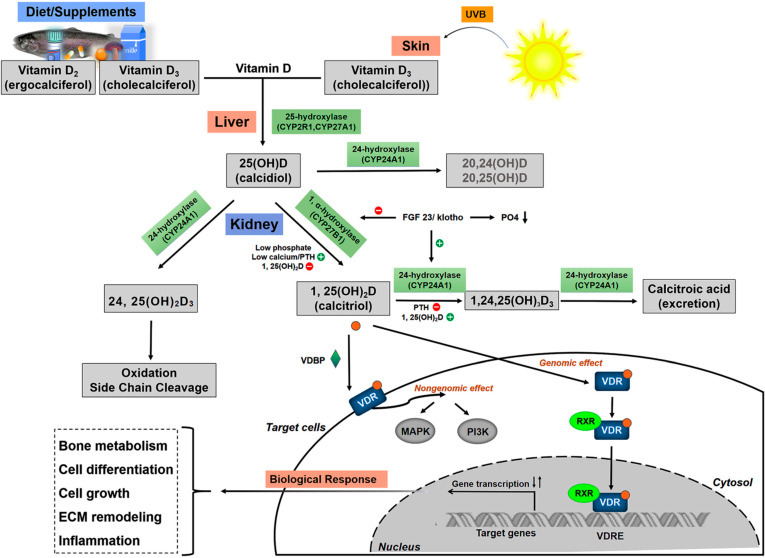
Metabolism of vitamin D and VD/VDR signaling. UVB, ultraviolet radiation b; FGF-23, fibroblast growth factor 23; PTH, parathyroid hormone; VDR, Vitamin D receptor; RXR, retinoid X receptor; VDRE, vitamin D responsive gene; ECM, extracellular matrix.

The local levels and activity of 1,25(OH)_2_D are mostly mediated by its catabolizing enzyme (CYP24A1) and VDR. 1,25(OH)_2_D can induce the expression of CYP24A1, the key vitamin D catabolizing enzyme found mostly in intestinal tissues. CYP24A1 catalyzes both 25(OH)D and 1,25(OH)_2_D via C23- or C24-hydroxylation pathways, forming 24,25(OH)_2_D_3_ and 1,24,25(OH)_3_D_3_ (or 1,23,25(OH)_3_D_3_) and initiating the inactivation of vitamin D for excretion (Figure 1). This negative feedback loop limits vitamin D overdosage through degradation of both 25(OH)D and 1,25(OH)_2_D [22,23,24]. The molecular basis of vitamin D signaling implies that the active metabolite 1,25(OH)_2_D binds to the transcription factor VDR with high affinity in the cytoplasm, forms a VD-VDR complex and induces VDR- mediated signaling transduction. Because VDR has been found to be nearly ubiquitously expressed, the effects of vitamin D induced gene activation affect almost every cell in the body. Upon activation by ligand binding, it facilitates the formation of the heterodimer of VDR with retinoid X receptor (RXR) in the nucleus, then binds to the specific vitamin D responsive elements (VDREs) and modulates up to one-third of all human genes, including those involved in the regulation of bone metabolism; cell-life processes (proliferation, differentiation, apoptosis); the immune system; oxidative stress; and lipid metabolism [25] (Figure 1). It has been suggested that both adequate vitamin D levels in the blood and the activity of VDR are crucial for the biological functions of vitamin D.

### 3.2. Biological Action of Vitamin D in Pancreatic Cells

Pancreatitis in both acute and chronic forms is initiated by injury to pancreatic acinar cells. The crosstalk between pancreatic acinar, ductal, and stellate cells and the immune system perpetuates an inflammatory response, resulting in localized pancreatic inflammation, systemic inflammation, or chronic disease. Emerging evidence suggests that vitamin D signaling can contribute to pancreatic homeostasis by exerting anti-inflammatory and antifibrotic activities. The effect of vitamin D may be supported by the expression of VDR and signaling in pancreatic cells. The role of vitamin D in the modulation of inflammatory processes has emerged from cellular studies (Table 1).

Pancreatic acinar cells are the major cell type in the pancreas. A previous study indicated that VDR levels are low in acinar cells of the human and rat pancreas [34,35]. In response to AP toxins (bile acids, alcohol, nicotine, etc.), trypsinogen activation within acinar cells triggers innate immune mechanisms that recruit immune cells (neutrophils are recruited initially, followed by macrophages, dendritic cells, and T cells) to the site of inflammation. Local injury is further exacerbated by the massive release of damaged-associated molecular patterns (DAMPs) from necrotic acinar cells that attract and activate immune cells, further prompting multiple inflammatory cascades and remote organ failure. VD/VDR has been shown to exhibit immunologic properties that regulate the immune response. The activation of toll-like receptor (TLR) and nuclear factor-kB (NF-κB) is essential for the initiation and progression of the systemic inflammatory cascade during pancreatitis. Vitamin D supplementation could downregulate TLRs in inflammatory diseases [36,37]. Studies have also demonstrated that 1,25(OH)_2_D reduces the nuclear translocation of NF-κB through its subunit p65, thereby inhibiting the activation of NF-κB and its downstream genes, including IL-8. Vitamin D acts as a negative modulator of TNF-α and IL-6 release, decreasing TNF-α, IL-6, and C-reactive protein (CRP) levels in pancreatitis [38]. Further studies are needed to confirm the underlying anti-inflammatory mechanisms of VD/VDR signaling in pancreatitis.

Abnormal activation of pancreatic stellate cells (PSCs), resulting from progressive necroinflammatory conditions of the pancreas, is the primary pathological feature of fibrosis in CP. In mice, among isolated pancreatic cells, only PSC and islet cells highly expressed VDR. Moreover, VDR expression in PSCs was five times greater than that in islet cells [35]. VDR plays a critical role in the development of CP because it attenuated inflammation and fibrosis in a cerulein-treated CP model, consistent with decreased PSC activation. Vitamin D_3_ has also been shown to initiate cellular differentiation and inhibit proliferation of cells from normal tissue.

## 4. Vitamin D and Pancreatitis in Clinical Studies

Serum 25(OH)D is a widely accepted biomarker to assess vitamin D status, owing to its stable concentration and half-life of 15–25 days [39]. There is no uniform international consensus that defines the deficiency, insufficiency, sufficiency, and toxicity of vitamin D [7,40,41,42,43]. The prevalence of vitamin D deficiency is overwhelming worldwide [44,45,46]. In developed countries, 36.8 to 40% of the population was moderately deficient in vitamin D (25(OH)D < 50 nmol/L), and 5.9–13% of the population was severely deficient (25(OH)D < 30 nmol/L) [47,48,49]. In China, the estimated prevalence of 25(OH)D levels < 50 nmol/L was reported to be 60% [50]. As pancreatitis is an inflammatory condition of the pancreas that leads to impairment of endocrine and exocrine function, disrupted absorption during disease or malnutrition due to prolonged fasting or exocrine function disorders can cause nutritional deficiencies. Acute recurrent pancreatitis and progression to CP are accompanied by extensive fibrotic tissue replacement and loss of exocrine pancreatic function during the course of the disease, leading to malabsorption and malnutrition over time [1,4]. Determining the serum levels of lipid-soluble vitamin D might be relevant for pancreatitis because of its dependence on photosynthesis in the skin as well as on direct intestinal resorption.

### 4.1. Vitamin D and Acute Pancreatitis

#### 4.1.1. Vitamin D Status in Patients with AP

Currently, a few studies have investigated the association between vitamin D and AP (Table 2). Decreased 25(OH)D levels were detected in cats and dogs with AP, which might be associated with calcium imbalance and mortality rates in animal AP [51,52]. Vitamin D deficiency/insufficiency is particularly common in patients with AP. One recent study using a large retrospective database of 36,087,380 patients between July 2014 and July 2019 found that patients with AP were more likely to develop vitamin D deficiency (odds ratio [OR]: 1.25, 95% confidence interval [CI]: 1.24–1.26, *p* < 0.0001); osteoporosis (OR: 1.89, 95% CI: 1.81–1.85, *p* < 0.0001), and fractures (OR: 1.58, 95% CI: 1.57–1.59, *p* < 0.0001) than those without AP [53]. Another study that reported the 25(OH)D levels on the admission of 73 patients with the first episode of AP showed that the prevalence of severe vitamin D deficiency (<13 nmol/L), deficiency (13–25 nmol/L), and insufficiency (26–50 nmol/L) was 23%, 20%, and 40%, respectively, while only 17% of patients had a normal level of 25(OH)D (>50 nmol/L) [38]. In a study of 242 AP patients in whom serum 25(OH)D levels were measured within 24 h of admission, 56.2% had vitamin D deficiency (≤25 nmol/L) and 28.5% had insufficiency (25–50 nmol/L), while only 15.3% of AP patients had normal vitamin D levels (>50 nmol/L) [12]. As baseline levels of vitamin D were not available and did not present the prevalence of osteoporosis, further studies should be conducted to assess vitamin D deficiency/insufficiency in AP patients in the future.

Another study reported that serum concentrations of 25(OH)D were statistically similar compared with the healthy control group, although patients in the pancreatitis group (mainly of the alcoholism as etiology) had markedly reduced [54]. However, the control group in this study was composed of a small sample size of 20 patients who were hospitalized for hernia repair. The study conclusions were not reliable, because the low vitamin D level found in the control group could be associated with the disease status.

#### 4.1.2. Imbalance of Vitamin D Metabolism as a Risk Factor for AP

##### Vitamin D Deficiency and Hypercalcemia-Mediated AP

Hypercalcemic states could be associated with the pathogenesis of AP [51]. Approximately 7–19% of patients with hypercalcemia develop pancreatitis owing to obstruction of the pancreatic duct by stones, activation of trypsin by excess calcium in the secretions, increased alkalinity which precipitates calcium, and vasculitis within the pancreas. Hyperparathyroidism, either primary or secondary to vitamin D deficiency, has been described in association with hypercalcemia-mediated by AP [57,58].

Although hypercalcemia seems to be the major risk factor, mutations in different genes have also been proposed. CYP24A1 mutations can cause vitamin D-mediated hypercalcemia and pancreatitis [59]. CYP24A1 deficiency contributes to unexplained vitamin D-mediated hypercalcemia or patients without baseline hypercalcemia or nephrocalcinosis [60].

##### Vitamin D_3_ Poisoning-Induced Pancreatitis

When an excess of dietary vitamin D is present, elevated systemic and local concentrations of 1,25(OH)_2_D can occur. The toxicity of the serum 25(OH)D concentration threshold differs from 50 ng/mL to 150 ng/mL [7,42]. When excess 1,25(OH)_2_D is present within a tissue, local hypervitaminosis D can be produced. Rare case reports have highlighted the possibility of increasing serum 25(OH)D concentrations into the normal range of single patients. Vitamin D intoxication was reported as the cause of hypercalcemic pancreatitis [61]. Several studies have demonstrated that AP could be caused by hypercalcemia following an excessive dose of vitamin D [62,63,64]. A case report described a patient in whom recurrent attacks of pancreatitis were induced by vitamin D poisoning associated with hypercalcemia. Vitamin D-induced AP reportedly occurred in a patient with a history of vitamin D_3_ supplementation and high levels of serum 1,25(OH)_2_D without other etiologies [65].

#### 4.1.3. Vitamin D Disorders Affect the Severity of AP

##### Vitamin D Levels Affect the Severity of AP

Although it remains unclear whether vitamin D deficiency is a cause or a consequence of severe disease, findings suggest that the degree of vitamin D deficiency may be related to disease prognosis. Serum vitamin D deficiency on admission was an independent risk factor for severe AP (OR: 5.37, 95% CI: 1.13–25.57, *p* = 0.015) and intensive care unit (ICU) admission (OR: 3.09, 95% CI: 1.24–7.69, *p* = 0.035) [12]. In the first two or three days of AP, serum vitamin D showed a significant drop and linear trend, which was related to alterations in the levels of CRP, a systemic inflammation marker. Nevertheless, CRP is not a prompt and precise indicator of the severity of AP, and the association of low levels of serum 25(OH)D at admission and parameters of organ failure have not been confirmed.

##### Gene Polymorphisms

There are studies on the relationship between VDR polymorphisms and the risk of AP. As vitamin D acts through VDR, impairment or reduced functions due to VDR gene polymorphisms are associated with the severity of AP [66,67]. This finding highlights the protective effect of VD/VDR signaling against AP. However, the mechanism of VD/VDR signaling in AP remains to be explored.

Vitamin D may suppress renin synthesis at the transcriptional level and influence renin-angiotensin system (RAS) activity, thus acting as a negative regulator of RAS. The association of AP and its severity with specific variants in key RAS/vitamin D pathway genes likely denotes a causal role for such systems in AP pathogenesis. The renin rs5707 G (rather than A) allele was associated with AP, infected necrosis, and mortality. The role of vitamin D/RAS in the pathogenesis or severity of AP needs further detailed analysis [68].

### 4.2. Vitamin D and Chronic Pancreatitis

#### 4.2.1. The Prevalence of Vitamin D Deficiency/Insufficiency in Patients with CP

CP is characterized by a progressive malabsorptive condition that affects the digestive and absorptive ability of the body, resulting in malnutrition over time [13]. Predominant factors leading to vitamin D deficiency are likely related to steatorrhea with malabsorption of vitamin D due to CP [69]. Additional risk factors for vitamin D deficiency include African–American ethnicity, reduced nutritional status, and diabetes mellitus, among others.

Numerous studies have assessed the prevalence of vitamin D deficiency/insufficiency in patients with CP (Table 3). A recent meta-analysis [70] including 12 studies reported that vitamin D deficiency (defined as <50 nmol/L) had the highest prevalence (57.6%, 95% CI 43.9–70.4) among fat-soluble vitamins (vitamins A, D, E, and K) in CP. Another study that included nine studies on the prevalence of vitamin D deficiency/insufficiency (<50 nmol/L, or <75 nmol/L) in 465 CP patients and 378 controls also indicated a high prevalence of vitamin D deficiency (65%) and insufficiency (83%) in CP patients [71]. The prevalence of vitamin D deficiency (<20 ng/mL) was 42% in a cohort of 147 patients with newly diagnosed CP [72].

#### 4.2.2. Vitamin D Deficiency/Insufficiency Associated with the Severity of Exocrine Function

An impaired exocrine pancreas function alters vitamin D metabolism. Based on structure, atrophy and ductal-related parameters in CP were associated with vitamin D deficiency [89]. The consequences of exocrine insufficiency, mostly indicated by elastase 1 levels in feces, might be relevant for serum levels of vitamin D_3_ [90,91]. As summarized in Table 3, pancreatic exocrine insufficiency varied in CP patients and correlated with vitamin D deficiency. Pancreatic enzyme replacement therapy improved vitamin D levels.

#### 4.2.3. CP-Related Osteopathy

Risk factors for CP include excessive alcohol consumption, cigarette smoking, and preconditions such as diabetes mellitus, exocrine pancreatic insufficiency, and anorexia. These only affect vitamin D synthesis and absorption but also impair bone metabolism, which could induce low bone mass density and subsequent disorders such as osteopenia and osteoporosis and a consequent increase in bone fragility and susceptibility to fractures [85]. A previous study reported that 5% of CP patients had osteoporosis, whereas 39% in total had osteopathy including osteopenia, osteoporosis, and osteomalacia [92]. As shown in Table 3, and according to a meta-analysis, osteoporosis occurs in about one-quarter of CP patients, and osteopenia or osteoporosis occurs in approximately two-thirds of CP patients. To prevent and cure osteoporosis and fractures as well as the accompanying morbidity, bone health screening should become an integral part of the medical and nutritional care for CP patients.

It has been reported that 25(OH)D is correlated with the severity of inflammation, fecal elastase (exocrine dysfunction), and bone mineral density (BMD) [93]. Another study reported that patients with CP had no correlation between BMD loss and duration of illness or vitamin D levels [94]. In tropic CP patients, vitamin D was diminished in nearly 90% of patients, but such an association is difficult to establish. Currently, there are no reports of BMD in other forms of CP with an early-onset, such as idiopathic CP or hereditary pancreatitis.

#### 4.2.4. CP-Related Diabetes

The development of diabetes mellitus in CP mainly occurs due to the destruction of islet cells by pancreatic inflammation. Loss of pancreatic islet cells occurs later in the disease process, as endocrine cells are diffusely distributed throughout the pancreatic parenchyma. Patients may develop type 3c (pancreatogenic) diabetes mellitus (T3cDM), which is complicated by concurrent decreased glucagon secretion [95]. From a biological perspective, vitamin D deficiency/insufficiency as a determinant of diabetes risk is plausible, given that both impaired insulin secretion and action have been reported with vitamin D insufficiency [6]. As 78.5% of T3cDM patients have CP, physicians must also be aware of the elevated risk of pancreatic cancer in this subset of patients. Measurement of serum 25(OH)D levels and supplementation in patients with T3cDM might therefore be beneficial. To our knowledge, there are no randomized controlled trials (RCTs) on this topic and they should be considered in future studies.

## 5. Vitamin D Supplementation and Its Analogs’ Potential in Pancreatitis

The dose of vitamin D supplementation for the prevention of vitamin D deficiency is still controversial. The Institute of Medicine (IOM) [96] and Scientific Advisory Committee on Nutrition (SACN) [41] recommend 600 IU or 400 IU daily to maintain serum 25(OH)D > 50 nmol/L. The Endocrine Society [7] suggests an intake of 1500–2000 IU daily in adults. The European Food Safety Authority (EFSA) [97] has set the upper limit for vitamin D supplementation for adults at 4000 IU per day. Another guideline focused on the pleiotropic effects of vitamin D recommends a target 25(OH)D concentration of 75 nmol/L, and age-, body weight-, disease-status, and ethnicity dependent vitamin D doses ranging between 400 and 2000 IU/day [98]. Supplementation should consider the specific aspects of their health outcome concerns, age, body weight, the latitude of residence, dietary and cultural habits, making the regional or nationwide guidelines more applicable in clinical practice.

Activated vitamin D helps maintain serum calcium levels by promoting the absorption of calcium in the upper small intestine and stimulating bone absorption by osteoclasts [99]. Studies have reported the benefits of vitamin D supplementation in both AP and CP. One multiethnic cohort study that enrolled 2810 pancreatitis patients (gallstone-related AP, *n* = 1210; AP not related to gallstones, *n* = 1222; recurrent AP or suspected CP, *n* = 378) showed associations between dietary factors and AP [100]. This study found that intake of vitamin D and milk were inversely associated with gallstone-related type, which indicated the importance of vitamin D intake in preventing AP, at least the gallstone-related type. Moreover, vitamin D supplementation in AP patients with vitamin D deficiency appeared to reduce the development of persistent organ failure [101]. Nevertheless, high-dose supplementation may cause excessive intestinal calcium absorption, and renal calcium and bone reabsorption [102,103,104,105], leading to hypercalcemia over time. Several studies have demonstrated that AP could be caused by hypercalcemia following an overdosage of vitamin D [61,62,63,64]. The appropriate dosage of vitamin D as a supplement in AP treatment remains to be determined. However, the role and use of vitamin D are not mentioned in any therapeutic guidelines for AP.

In an RCT [106] of 27 patients, compared to ultraviolet ray placebo (weekly tanning bed sessions), daily cholecalciferol supplements (1520 IU) increased serum 25(OH)D levels in patients. Another RCT demonstrated that compared with 300,000 IU, a single dose of 600,000 IU intramuscular vitamin D_3_ was a more effective form of vitamin D supplementation over six months in CP patients [88]. A systematic review and meta-analysis of RCTs about nutritional management of CP indicated the substantial effect of vitamin D supplementation in CP [107]. Several practice guidelines recommend that patients with CP should have periodic evaluations for malnutrition, including tests for osteoporosis and fat-soluble vitamin deficiency. When fat-soluble vitamins are insufficient, vitamin D should be supplemented appropriately [13,14,15,17,108,109].

Currently, little data is available on the dosage and type of vitamin D supplementation in pancreatitis patients, as well as the magnitude of the benefits obtained. None of the guidelines provide clear and consistent recommendations about the dosage, route of administration, or type of vitamin D supplementation. Pancreas specialists, but not general physicians, were more likely to advise vitamin D testing and vitamin supplementation. Therefore, health education for physicians should be enhanced to address this situation [110].

Despite treatment with high doses of vitamin D, low levels persist. High and systemically administered doses are needed to achieve antiproliferative effects for treatment with vitamin D_3_, with a risk of hypercalcemia and hypercalciuria. When treated with vitamin D_3_, high doses and systemic administration are required to achieve anti-proliferative effects, with the risk of hypercalcemia and hypercalciuria. Vitamin D analogs have been developed with fewer hypercalcemic effects and without affecting cell proliferation. More than 3000 vitamin D analogs have been synthesized to enhance VDR binding affinity and increase metabolic stability, but few are clinically approved. Experimental studies have shown that calcipotriol, a potent and nonhypercalcemic vitamin D analog, could control VDR induction and attenuate inflammation and fibrosis in a cerulenin-treated CP model, consistent with decreased PSC activation [28]. Similar results showed that vitamin D_2_, vitamin D_3_, and calcipotriol significantly reduced the expression of α-SMA in freshly isolated PSCs without full activation [26]. Calcipotriol suppresses pancreatitis and enhances pancreatic cancer therapy by modulating transforming growth factor β (TGFβ), the main profibrogenic cytokine that drives fibrosis during CP [28]. Vitamin D inhibits ethanol metabolism, or antioxidants in alcoholic pancreatitis may arise in part through their ability to attenuate connective tissue growth factor (CCN2) production by mouse PSC [111]. We believe that the above studies shed light on the potential use of vitamin D in the treatment of CP.

## 6. Conclusions and Perspective

Studies have demonstrated that vitamin D plays a critical role in the regulation of inflammation in AP and CP, where local and systemic inflammation and alterations in long-term metabolic status are key elements. Numerous cross-sectional, cohort and longitudinal studies have shown an association among 25(OH)D levels, inflammation, osteopathia, and glucose metabolism in pancreatitis. Interventional studies have failed to demonstrate an unequivocally beneficial effect of vitamin D supplementation in pancreatitis, but some encouraging results have emerged from trials on patients at risk of developing complications related to pancreatitis. The included studies have some limitations: (1) most reported single observations; (2) nearly all studies were underpowered given the small sample size; (3) most studies did not report medications at baseline or during the study; and (4) different doses and types of vitamin D were administered, which may be metabolized differently and provide either no benefit or result in an unfavorable benefit/risk ratio. VD/VDR signaling induced anti-inflammatory and anti-fibrotic effects in pancreatitis, and further research is required to detail the underlying molecular mechanisms. Vitamin D and its analogs have shown promising potential during inflammatory and fibrotic diseases, but greater focus is required to carry out well-designed RCTs of AP and CP to clinically evaluate these treatment methods.

In conclusion, despite the available evidence of a connection among signaling pathways of vitamin D, inflammatory cytokines and pancreatitis, the current data are insufficient to demonstrate a general causal role of vitamin D deficiency in the pathogenesis of pancreatitis, or a therapeutic role for its supplementation in pancreatitis. The study of vitamin D and pancreatitis is still in its infancy. Long-term, well-designed, interventional clinical trials should be conducted to achieve a better understanding of the therapeutic potential of supplementation in patients with pancreatitis with vitamin D deficiency with regard to doses, duration of therapy, side effects, and short-term, and long-term results. In fact, we believe that vitamin D deficient patients at risk of developing CP-related complications are the most promising target populations for supplementation. Patients with CP should receive optimal preventive care, and more physicians should be better informed to provide optimal vitamin D testing and offer bone health surveillance. The dose and duration should be decided considering vitamin D deficiency/insufficiency and age.

## Figures and Tables

**Table 1:** VD/VDR signaling in pancreatic cells and its biological actions.

Pancreatic Cells	VDR Expression	Vitamin D Induced Targets Expression	Biological Actions
Pancreatic stellate cells [26,27,28]	High	IL-6, Collagen I, α-SMA and fibronectin↓	Inhibitory effects against proliferation and fibrosis in vitro or in chronic pancreatitis models
Islets cells [29,30,31]	Low	VDR, CYP24A1, CaSR↑	1,25 Dihydroxyvitamin D_3_ has a direct and genomic action on β-cell functions including insulin secretion; in CP patients, the highest CYP24A1 levels were found in the endocrine cells.
Pancreatic acinar cells [31]	Absent or low basal level	VDR, CYP24A1, CaSR↑	CYP24A1 is increased both during inflammation (as in chronic pancreatitis) and during malignant transformation (as in pancreatic ductal adenocarcinoma)
Pancreatic ductal cell [32]	Low	Increased VD-induced VDR, CDKN1A, CDK1 expression↑, high-dose VD downregulated VDR expression	Promoting the cell cycle of normal ductal cells
Pancreatic progenitor cells [33]	VDR expressing in the nucleus, cytoplasm, and plasma membrane	VD-induced VDR expression↑	Promote cell viability and proliferation.

α-SMA, α-smooth muscle actin; CaSR, calcium-sensing receptor; CDKN1A, cyclin-dependent kinase inhibitor 1A; CDK1, cyclin-dependent kinase; Upward arrow (↑) signifies an increase above normal due to vitamin D induction; Downward arrow (↓) signifies a decrease below normal due to vitamin D induction.

**Table 2:** Prevalence of Vitamin D Deficiency/insufficiency in Patients with AP.

Author, Year	Study Design	Country	AP Patients (*n*)	Etiology of AP (%)	Vitamin D Deficiency (*n*, %)	Osteoporosis (*n*, %)
Abou Saleh et al., 2020 [53]	Retrospective cohort study	USA	196,080	NA	Deficiency (17.7)	17,120 (8.7)
Bang et al., 2011 [55]	Prospective cohort study	England	73	Gallstones (52), Alcohol consumption (30), Idiopathic (11), Alcohol and gallstone (3), Other (4)	severe deficiency <13 nmol/L (23) deficiency 13–25 nmol/L (20) insufficiency 26–50 nmol/L (40)	NA
Huh et al., 2019 [12]	Prospective cohort study	Korea	242	Gallstones (52.5), Alcohol consumption (36), Hypertriglyceridemia (5), Idiopathic (6.6)	Deficiency < 10 ng/mL (56.2) Insufficiency 10–20 ng/mL (28.5)	NA
Leerhøy et al., 2018 [56]	Prospective cohort study	Denmark	29	Post-ERCP (100)	Insufficiency < 50 nmol/L (34.5)	NA

NA, not available.

**Table 3:** Prevalence of vitamin D deficiency/insufficiency in patients with chronic pancreatitis.

Study	Patients	Sample Size	Age, Years *	Etiology (%)	PEI (%)	PERT (%)	EI (%)	Osteopathy (%)	Serum 25(OH)D Deficiency
Observational Studies (Cross-Sectional Studies)									
Olese et al., 2017, Denmark [72]	CP	147	NA	NA	NA	NA	NA	NA	42% (<50 nmol/L)
Tang et al., 2021, China [73]	CP	104	46.1 (14.4)	Idiopathic, 68.3 Tropical alcoholic 31.7	27.9	49.0	26.9	Osteopenia, 30.8; Osteoporosis, 5.8	73% (<20 ng/mL)
Joker-Jensen et al., 2020, England [74]	CP	115	57.9 (13.0)	Alcoholic, 50 Tropical, NA Idiopathic, NA	60.8	35.6	37.4	NA	22% (<25 nmol/L)
Stigliano et al., 2018, European (multicenter) [75]	CP	211	60	Alcoholic 43.60 Idiopathic 18.95 Hereditary 4.26 Obstructive 5.68 Other 27.48	56.42	54.97	37	Osteopenia 42.18; Osteoporosis 21.80	56.37% (<20 ng/mL)
Min et al., 2018, USA [76]	CP	91	48.6 (10.4)	Toxic/metabolic 59.3 Idiopathic 18.7 Genetic 14.3 Autoimmune 5.8 Obstructive 2.2	84.6	NA	NA	Osteopenia 46.7; Osteoporosis 22.2	62.50%
Kumar et al., 2017, India [77]	CP	102	40.8 (12.6)	Alcoholic 67 Tropical 35	NA	NA	NA	Osteomalacia and low bone mass 36	67.6% (<30 ng/mL)
Pezzilli et al., 2015, Italy [78]	CP	30	57.0 (13.1)	NA	56.7	NA	23.3	NA	86.6% (<20 ng/mL)
Sikkens et al., 2013, Holland (Prospective) [79]	CP	40	52 (11)	Alcoholic 50 Idiopathic 43 Other 7	70	48	45	Osteopenia 45; Osteoporosis 10	53% (<38 pmol/L)
Klapdor et al., 2012, Germany (Prospective) [80]	CP	37	NA	NA	NA	100	NA	NA	86.5% (<30 ng/mL), 37.8% (<10 ng/mL)
Dujsikova et al., 2008, Czech Republic [81]	CP	73	46 (13)	Alcoholic 11 Idiopathic 89	NA	NA	NA	Osteopathy 39; Osteopenia 26; Osteoporosis 5; Osteomalacia 8	86.3% (<75 nmol/L)
Prospective Case—Control Study									
Duggan et al., 2015, Ireland [82]	CP	29	44.3 (12.3)	Alcoholic 62.1 Idiopathic 27.6 Other 10.3	NA	NA	NA	Osteoporosis 31; Osteopenia 44.8	48.3% (<30 nmol/L)
	Controls	29	45.8 (9.8)	NA	NA	NA	NA	Osteoporosis 6.9; Osteopenia 51.7	20.7% (<30 nmol/L)
Duggan et al., 2014, Ireland [83]	CP	62	47.9 (12.5)	Alcoholic 38.7	34.8	NA	NA	NA	58% (<20 ng/mL)
	Controls	66	47.7 (11)	NA	NA	NA	NA	NA	61.7%
Prabhakaran, et al., 2014, India [84]	CP	103	38.6 (20.6)	Alcoholic 70 Idiopathic 29.1 Post-traumatic 0.9	20.4	NA	37.8	Osteoporosis 30.1; Osteopenia 39.8	19.4% (<10 ng/mL)
	Controls	40	36.7 (20.7)	NA	NA	NA	NA	NA	38.59 ± 26 ng/mL *
Duggan et al., 2012, Ireland [85]	CP	62	47.9 (12.5)	Alcoholic 38.7 Other 61.3	NA	NA	NA	Osteoporosis 34; Osteopenia 39.6	47.5 ± 21.6 mmol/L *
	Controls	66	47.74 (11)	NA	NA	NA	NA	Osteoporosis 10.2; Osteopenia 33.9	46.4 ± 20.4 mmol/L *
Joshi et al., 2011, India [86]	CP	72	31.1 (10.3)	Tropical calcific pancreatitis	46	46	72	The BMD Z-scores at the lumbar spine −1.0 ± 1.0 total hip −1.2 ± 1.2	86% (<50 nmol/L)
	Controls	100	32.6 (9.6)	NA	NA	NA	NA	NA	85%
Sudeep et al., 2011, India [87]	CP	31	35.8 (9.0)	Tropical fibro calculous pancreatitis 65 Idiopathic 35	69	0	68	Osteoporosis 29	52% (<20 ng/mL)
	Controls	35	38.6 (5.2)	NA	NA	NA	NA	Osteoporosis 9	24%
Mann et al., 2003, Germany [11]	CP	42	52.6 (13.5)	NA	78.5	NA	NA	DEXA Ward’s trangle (WARD) 92.2% ± 5.2%	26.7 ± 9.7 nmol/L *
	Controls	20	48.9 (6.4)	NA	NA	NA	NA	DEXA WARD 97.1% ± 3.1%	69.5 ± 13.5 nmol/L *
Double Blinded, Randomized Controlled Trial									
Reddy et al., 2013, India [88]	CP	40	33 (9)	Tropical Calcific (idiopathic)	NA	52.5	92.5	NA	40% (25–50 nmol/L) 72% (<25 nmol/L)

CP, chronic pancreatitis; PEI, pancreatic exocrine insufficiency; EI, endocrine in-sufficiency; PERT, pancreatic enzyme replacement therapy; NA, not available; DEXA, dual-energy X-ray absorptiometry. * Data presented as mean ± SD.

## Data Availability

No new data were created or analyzed in this study. Data sharing is not applicable to this article.
